# Rates, costs, return to work and reoperation following spinal surgery in a workers’ compensation cohort in New South Wales, 2010–2018: a cohort study using administrative data

**DOI:** 10.1186/s12913-021-06900-8

**Published:** 2021-09-11

**Authors:** AM Lewin, M Fearnside, R Kuru, BP Jonker, JM Naylor, M Sheridan, IA Harris

**Affiliations:** 1 South Western Sydney Clinical School, UNSW; Whitlam Orthopaedic Research Centre, Ingham Institute for Applied Medical Research, NSW Liverpool, Australia; 2 Neurosurgery, Westmead Hospital, Westmead, Australia; 3 Lake Macquarie Medical Centre, Gateshead, Australia; 4 Royal Prince Alfred Hospital, Institute of Academic Surgery, NSW Camperdown, Australia; 5 Department of Neurosurgery, Liverpool Hospital, Liverpool, Australia

**Keywords:** Spine, Spinal, Surgery, Workers’ compensation, Outcomes, Cost, Return to work, Reoperation, Elective, Fusion, Decompression

## Abstract

**Background:**

Internationally, elective spinal surgery rates in workers’ compensation populations are high, as are reoperation rates, while return-to-work rates following spinal surgery are low. Little information is available from Australia. The aim of this study was to describe the rates, costs, return to work and reoperation following elective spinal surgery in the workers’ compensation population in New South Wales (NSW), Australia.

**Methods:**

This retrospective cohort study used administrative data from the State Insurance Regulatory Authority, the government organisation responsible for regulating and administering workers’ compensation insurance in NSW. These data cover all workers’ compensation-insured workers in New South Wales (over 3 million workers/year). We identified a cohort of insured workers who underwent elective spinal surgery (fusion or decompression) between January 1, 2010 and December 31, 2018. People who underwent surgery for spinal fracture or dislocation, or who had sustained a traumatic brain injury were excluded. The main outcome measures were annual spinal surgery rates, cost of the surgical episode, cumulative costs (surgical, hospital, medical and physical therapy) to 2 years post-surgery, and reoperation and return-to-work rates 2 years post-surgery.

**Results:**

There were 9343 eligible claims (39.1 % fusion; 59.9 % decompression); claimants were predominantly male (75 %) with a mean age of 43 (range 18 to 75) years. Spinal surgery rates ranged from 15 to 29 surgeries per 100,000 workers per year, fell from 2011-12 to 2014-15 and rose thereafter. The average cost in Australian dollars for a surgical episode was $46,000 for a spinal fusion and $20,000 for a decompression. Two years post-fusion, only 19 % of people had returned to work at full capacity; 39 % after decompression. Nineteen percent of patients underwent additional spinal surgery within 2 years of the index surgery, to a maximum of 5 additional surgeries.

**Conclusion:**

Rates of workers’ compensation-funded spinal surgery did not rise significantly during the study period, but reoperation rates are high and return-to-work rates are low in this population at 2 years post- surgery. In the context of the poor evidence base supporting lumbar fusion surgery, the high cost, increasing rates, and the increased likelihood of poor outcomes in the workers’ compensation population, we question the value of this procedure in this setting.

**Supplementary Information:**

The online version contains supplementary material available at 10.1186/s12913-021-06900-8.

## Background

There is little high-quality evidence supporting the effectiveness of spinal fusion surgery for most indications; spinal decompression surgery also has limited evidence [1, 2]. Related to this lack of evidence, practice variation for spinal surgery is high in Australia. Practice or geographic variation in surgery refers to different rates of surgical procedures between comparable populations. Unwarranted variation arises where there is uncertainty or a lack of evidence regarding the effectiveness or clinical indications for a surgery. The geographic areas with the highest rates of lumbar fusion perform surgery at a rate seven times that of the areas with the lowest rates; this difference is five-fold for lumbar decompression. This geographic variation may reflect low-value care that is not evidence based [3]. In addition, there is considerable between-surgeon variability in surgical technique and indications for many spinal procedures [4]. Internationally, rates of spinal surgery are increasing [5–12]. In Australia, the rate of lumbar fusion performed in the private sector increased by 175 % between 1997 and 2006 [6]. In 2012-13, the total cost of spinal fusion in Australia was AUD 650 million for approximately 14,000 surgeries, making it the most expensive reimbursed procedure in terms of cost per surgery and the fourth highest overall cost behind knee and hip replacements and childbirth [3]. Internationally, reoperation rates are high and return-to-work rates are low [5, 13–15].

Outcomes after spinal surgery are worse in the Workers’ Compensation-insured population compared to non-compensated groups [16, 17]. Authors of a 2012 study of 476 patients treated under workers’ compensation in NSW found low return-to-work rates, and high rates of ongoing physiotherapy and opioid use two years after lumbar spinal surgery [13]. Otherwise, little work has measured the use and outcomes of elective spinal surgery in the Workers’ Compensation population in Australia.

The aim of this study was to provide an accurate measure of the rates, costs, return to work and reoperation following elective spinal surgery in a population of insured workers in NSW, Australia from 2010 to 2018.

## Methods

### Study design, setting and participants

For this retrospective cohort study, we used administrative data from the State Insurance Regulatory Agency (SIRA) to identify all adult workers (aged ≥ 18 years) who underwent workers’ compensation-funded elective spinal surgery in the Australian state of New South Wales (NSW) between January 1, 2010 and December 31, 2018, with follow-up to December 31, 2018. We excluded workers who sustained a traumatic brain injury as well as those with any missing cost or surgical data. To identify elective surgeries, we excluded claims for fracture or dislocation as they indicate trauma (Supplemental Table S1). This research involved deidentified administrative individual-level patient data, and was performed in accordance with the Declaration of Helsinki; ethical approval was granted by the South Western Sydney Local Health District Human Research Ethics Committee (HREC/16/LPOOL/189; LPN HE16/097).

### Data Source

SIRA (www.sira.nsw.gov.au) is the government organisation responsible for regulating and administering workers’ compensation insurance in NSW and for ensuring that people who experience workplace injuries have access to treatment. Databases held by SIRA capture injury date, nature and bodily location of injury; type, cost and date of treatment received and work status for all claims; data are updated regularly by the insurer until the claim is closed. No other data sources were used in this study. Treatment type includes billing by medical professionals using the Australian Medical Association (AMA) List of Medical Services and Fees and items for medical and allied health services (e.g. physiotherapy) specific to SIRA. Work status is recorded monthly by the insurer while a claim remains open and identifies whether a claimant is working at full capacity, working at reduced capacity, not working, retired or deceased. An internal audit of data quality found 97 % of surgical items were billed appropriately.

### Case ascertainment

Using item numbers from the AMA fees list we identified fusion, disc replacement and decompression surgery (Supplemental Table S2). We used a hierarchical definition for surgery type: any surgery including fusion (i.e., fusion with or without disc replacement and/or decompression) was classified as a fusion; a disc replacement with or without decompression was classified as a disc replacement; and decompression alone was classified as decompression. We identified spine region (cervical, lumbar), using item descriptions in the AMA fees list and the ‘bodily location of injury’ variable captured by SIRA (Supplemental Table S3).

### Rates, costs and outcomes

SIRA supplied denominator data on the total number of insured workers in NSW by fiscal year, allowing calculation of spinal surgery rates.

We identified four cost categories: direct surgical costs (surgical item numbers from the AMA Fees List); medical treatment (pharmacy, anaesthesia, imaging, pathology, specialist consultations, diagnostic procedures, intensive care unit procedures, general practitioner services, public hospital medical reports/health records, surgical assistant); hospital costs (private/public bedstay, private operating theatre fees, surgeon instrument fees, surgical items, other therapies/treatments (prosthesis fees)); and physical treatment (chiropractic, exercise physiology, rehabilitation, osteopathy, physiotherapy, massage, other allied health). Total episode-of-surgery cost comprised surgical billing plus medical and hospital costs; costs calculated up to two years also included physical treatment costs.

We defined return-to-work status at two years as the work status nearest to but not exceeding the 24-months-post-surgery date. We defined reoperation as any spinal surgery with a surgery date after the initial spinal surgery. People with > 1 open claim at the time of surgery were excluded from the return- to-work analysis as it could not be determined which of the claims was associated with work status.

Ascertainment of cumulative cost, return-to-work and reoperation outcomes at 24 months post-surgery was restricted to index surgeries at least 24 months prior to the end of the study period (December 31, 2018) to allow at least 2 years between spinal surgery and the end of follow-up.

### Statistical methods

Characteristics of the study cohort are presented as means/medians or proportions, as appropriate. Surgery rates were calculated as the number of surgeries in a given fiscal year divided by the insured population for that year, presented per 100,000 insured workers. We presented mean costs (Australian dollars [AUD]) to describe cost to the workers’ compensation system. Reoperation and return-to-work outcomes are presented as proportions. Analyses to 24 months (cumulative costs, return to work and reoperation) are restricted to surgeries at least 24 months prior to study end date to allow adequate time for outcome ascertainment. All analyses were performed using Stata, version 15 [18].

## Results

### Study Population

**Fig. 1:**
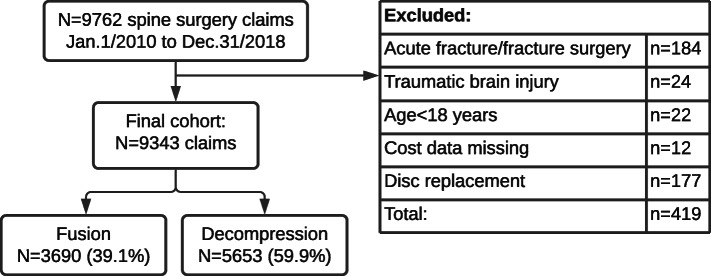
Study cohort

We used data from 9,762 spinal surgery claims (any spine region) under the workers’ compensation scheme in NSW that were active between January 1, 2010 and December 31, 2018. We excluded 242 claims (n = 184 acute fracture/dislocation; n = 24 traumatic brain injury; n = 22 aged under 18 years; and n = 12 with missing/incomplete data), leaving 3,690 (38.8 %) fusion (with or without decompression), 177 (1.9 %) disc replacement and 5,653 (59.4 %) claims for decompression alone (Fig. 1). Due to low disc replacement numbers, this analysis focuses on fusion and decompression, for a total of 9,343 claims. The cohort was predominantly male, comprising 72 % of fusion and 77 % of decompression claims. The mean age was 43 (range 18 to 75) years; 91 people (~ 1 %) were over 65. Median time from injury to surgery was 26 months for fusion and 9 months for decompression. Claims remained open for a mean 3.3 years after spinal surgery, with individual follow-up times ranging from 1 month to 9 years. In the 31-day period preceding surgery, 33 % of fusion and 40 % of decompression claimants were working (in either full or reduced capacity) though pre-operative work status was only available for 83 % of the cohort (Table 1).

**Table. 1:** Characteristics of the study cohort by surgery type

	Fusion	Decompression
**Overall**	***N* = 3690 claims**	***N* = 5653**
Mean (SD) age, years	43 (10)	43 (11)
Male, n (%)	2650 (71.8)	4353 (77)
Median (IQR) time to surgery, months	26 (10, 63)	9 (4, 25)
Mean (SD) follow-up time, years	3.5 (2.1)	3.2 (2.2)
Working prior to surgery, n/N (%)^a^	1036/3183 (32.5)	1796/4538 (39.6)

*SD* standard deviation; *IQR* interquartile range

^a^ Work status measured in the 31-day period prior to surgery; pre-operative work status was available for 86.2 % (3183/3690) of the fusion cohort and 80.3 % (4538/5653) of the decompression cohort

### Surgery rates

**Fig. 2:**
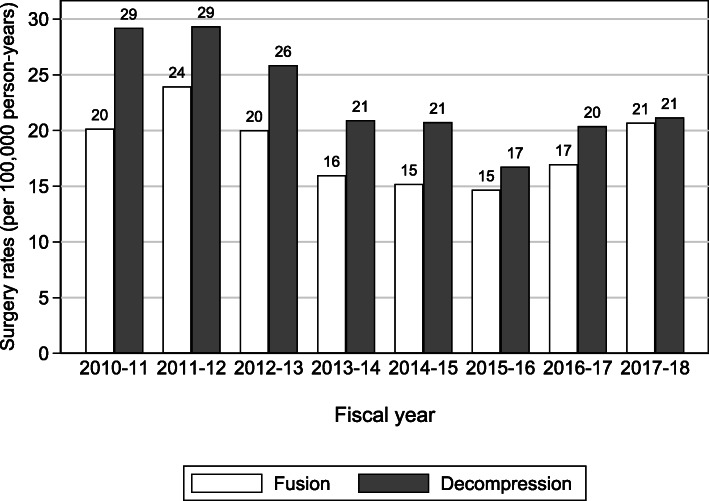
Rates of spinal fusion and decompression surgery by fiscal year, 2010-11 to 2017-18

Between fiscal years 2011-12 and 2015-16, spinal fusion and decompression rates declined by 39 % (from 29 to 17 procedures per 100,000 person-years) and 43 % (from 24 to 15 procedures), respectively. Rates rose in the subsequent two years to fiscal 2017-18: fusion by 41 % and decompression by 26 % (Fig. 2).

### Surgical, medical, hospital and physical treatment costs

**Fig. 3:**
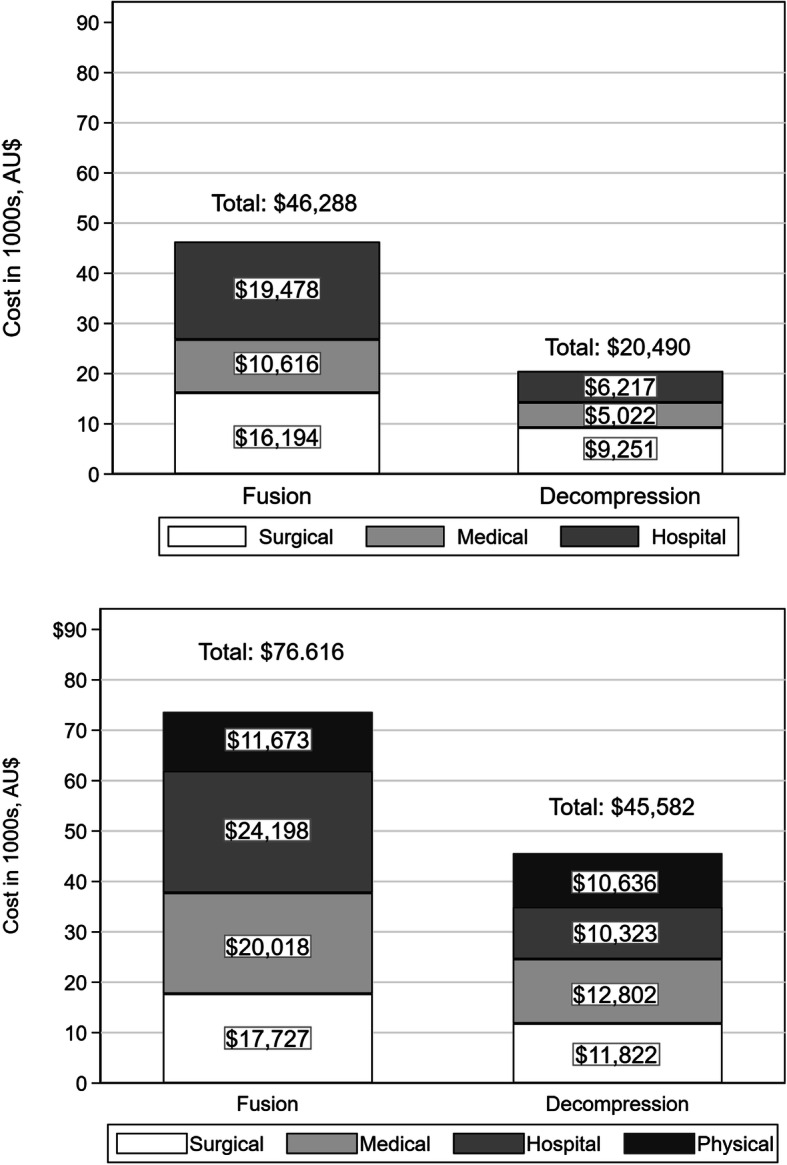
Mean cost per patient for surgical episode (top) and cumulative costs to 24 months (bottom) by surgery type, Australian dollars

The mean (standard deviation [SD]) total cost of a single episode of surgery, including surgeon, hospital and medical costs, was $46,288 (SD 22,112) for fusion and $20,490 (SD 6,843) for decompression. Two years post-surgery, the mean cumulative surgical, hospital, medical and physical costs increased by 59 % to $74,560 (SD 42,915) for fusion and by 122 % to $45,487 (SD 32,501) for decompression. Physical treatment such as physiotherapy and rehabilitation added $11,069 and $10,576 for fusion and decompression, respectively (Fig. 3).

### Reoperation rates

Reoperation rates were calculated for index surgeries that occurred to December 31, 2016 to allow at least 2 years between spinal surgery and the end of follow-up. Two years after fusion surgery, 18 % (511/2,841) of people had undergone at least one additional spinal surgery, to a maximum of 5 reoperations. Reoperation was 20 % (906/4,485) following decompression surgery, with a maximum of 4 additional surgeries within two years.

### Return to work rates

**Fig. 4:**
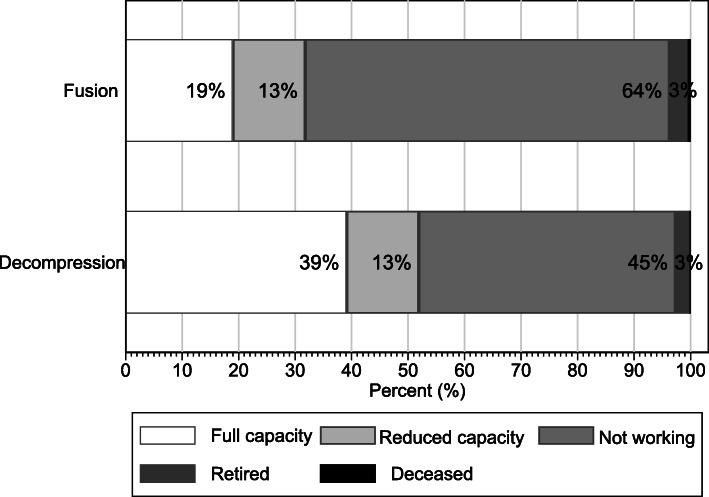
Return-to-work status at 24 months post-surgery by surgery type

Only 19 % of people were working at full capacity by two years post-surgery following fusion, compared to 39 % following decompression. For both surgery types, an additional 13 % were working at reduced capacity at two years. Approximately 3 % of claimants retired or died and thus were ineligible to return to work. The proportion of people not working in any capacity at two years was 64 % following fusion and 45 % after decompression (Fig. 4; Supplemental Table S4).

### Outcomes by spine region

**Fig. 5:**
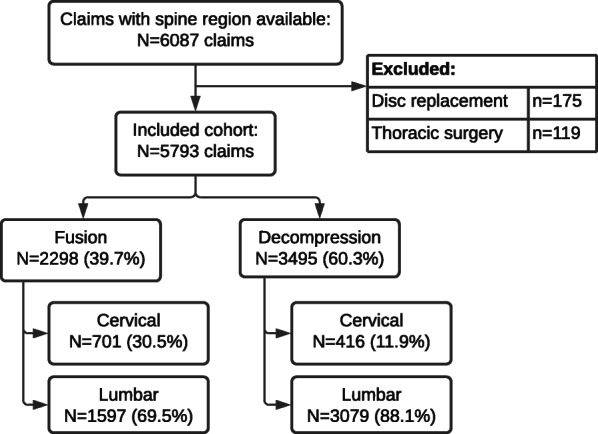
Study cohort with spine region data available

We were able to establish region of surgery (cervical, thoracic or lumbar) for 5,793 claims (62.0 % of the included cohort; Fig. 5). Due to low numbers, we excluded thoracic spinal surgery (n = 119; 1.3 % of the original eligible cohort) from this analysis. People undergoing cervical surgery were slightly older than those undergoing lumbar surgery, with a mean age of 45.0 versus 41.2 years, respectively, for fusion, and 45.7 versus 41.6 for decompression. Most spinal surgery patients were male, regardless of spine region. Median (interquartile range [IQR]) time from injury to surgery was shortest for lumbar decompression at 7.8 (4.0, 21.8) months, and longest for lumbar fusion, at 32.3 (14.2, 82.3) months (Table 2).

**Table. 2:** Characteristics of the study cohort by surgery type and spine region

	Fusion* N* = 2298	Decompression* N* = 3495
**Cervical**	***N* = 701**	***N* = 416**
Mean (SD) age, years	45 (8.8)	45.7 (9.3)
Male, n (%)	476 (67.9)	333 (80)
Median (IQR) time to surgery, months	14.7 (5.8, 35.5)	10.2 (4.6, 25.4)
Mean (SD) follow-up time, years	3.3 (2.1)	3.2 (2.2)
Working prior to surgery, n/N (%)^*^	233/581 (40.1)	153/323 (47.3)
**Lumbar**	***N* = 1597**	***N* = 3079**
Mean (SD) age, years	41.2 (10.3)	41.6 (11.4)
Male, n (%)	1176 (73.6)	2400 (77.9)
Median (IQR) time to surgery, months	32.3 (14.2, 82.3)	7.8 (4, 21.8)
Working prior to surgery, n/N (%)^a^	435/1412 (30.8)	985/2473 (39.8)

*SD* standard deviation; *IQR* interquartile range

^a^Work status measured in the 31-day period prior to surgery; pre-operative work status was available for 81 % of the cohort with spine region available

**Fig. 6:**
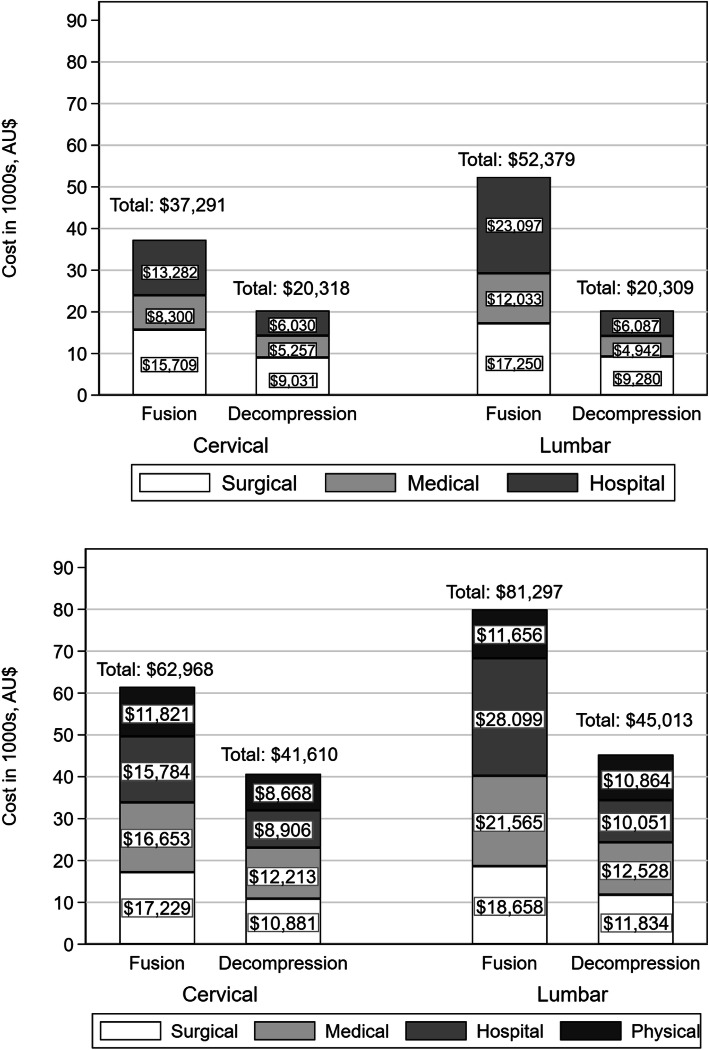
Mean cost per patient for surgical episode (top) and cumulative costs to 24 months (bottom), by surgery type and spine region

**Fig. 7:**
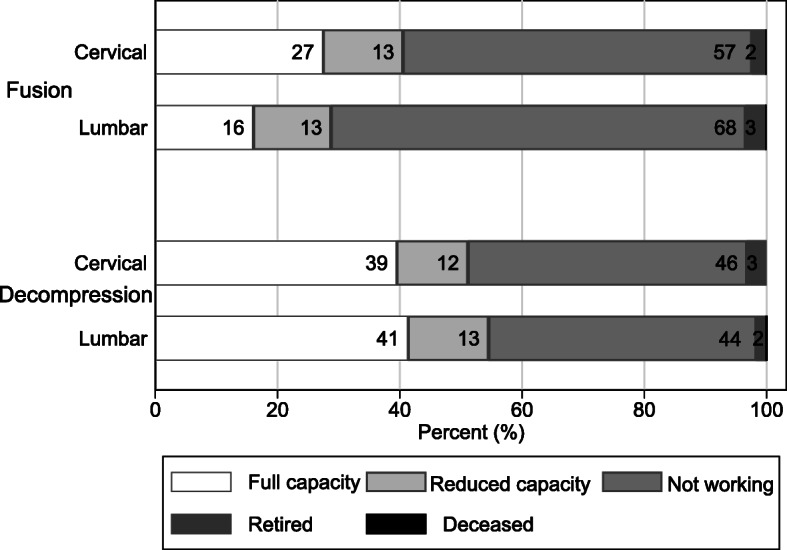
Return-to-work status at 24 months by surgery type and spine region

Results by spine region broadly mirrored those seen in the overall cohort, though lumbar fusion was associated with higher costs, higher reoperation rates and lower return-to-work rates than cervical fusion. There was little difference between cervical and lumbar decompression in terms of costs (Fig. 6), reoperation (Supplemental Figure S1) or return to work (Fig. 7)

## Discussion

In this study of the workers’ compensation population in NSW, annual spinal surgery rates ranged from 15 to 29 surgeries per 100,000 workers, depending on surgery type, increasing modestly since 2015. Within two years of surgery, nearly one in five people had received at least one reoperation, and 64 % of people had not returned to work in any capacity after spinal fusion.

Our results are similar to those seen in prior research. Spinal surgery rates have been increasing globally, both in the workers’ compensation and general populations, with the greatest increase seen in people over age 65, a group who comprise only a small proportion of those undergoing surgery insured by workers’ compensation [5, 8, 19]. It is difficult to ascribe a clinical or demographic reason for the decline to 2015 and subsequent rise in spinal surgery rates in NSW during the study period, though 2015 marked the introduction of structural reforms in the administration and delivery of workers’ compensation in NSW. This included the establishment of the State Insurance Regulatory Authority - an independent insurance regulator - and the creation of a new nominal insurance provider to deliver the State’s insurance schemes [20]. This changed regulatory environment, combined with a lack of clinical practice guidelines for back pain and high practice variation in elective spinal surgery may explain at least some of the variability in spinal surgery rates over the study period.

The Second Australian Atlas of Healthcare Variation (the Atlas) reported annual lumbar fusion rates (per 100,000 population) of 26 nationwide and 27 (range 12 to 47 by local area) in NSW from 2012-13 to 2014-15. [21]. Another study reported lumbar fusion rates of 23.1 in 2006 in private hospitals in Australia and 20.5 for NSW, with an additional 5.2 lumbar fusion surgeries performed in public hospitals in NSW.[6] These are comparable to fusion rates reported in our study, which ranged from 15 to 24 (per 100,000 workers) annually between 2010 and 2018. The Atlas reported decompression rates of 81 nationwide, and 85 (range 45 to 156 by local area) in NSW, substantially higher than our results, which ranged from 17 to 29 decompression surgeries per 100,000 workers. There are a few possible explanations. The rates of surgery reported in our study represent a subset of those reported in the Atlas, which includes public, private and workers’ compensation-funded surgeries. The Atlas counted surgeries rather than individuals, whereas our study reported on individuals as the unit of analysis; given the high reoperation rate, this may explain some of the disparity. Finally, the highest rates and biggest increases in lumbar surgery occur in the population outside of that considered in this study, people above age 65, whereas only 1 % of our cohort was over 65. In Norway between 1999 and 2013, lumbar decompression surgery increased 5-fold among people over 75 years, reaching 167.8 surgeries per 100,000 in 2013 [5]. Similar findings have been reported in the US [10, 19]. In a report on all spinal surgeries (~ 31,000) undertaken in Japan in 2011, the most frequent age group was 70–79 years, and 63.1 % of the cohort was over 60 years of age in 2011, compared to 49 % in 2001. Unfortunately, the Atlas does not report age-specific rates, making a direct comparison impossible.

The cost of an episode of lumbar fusion in this study was AUD 52,379, slightly higher than the AUD 43,000 reported by the Australian Commission on Safety and Quality in Health Care in 2016 [3]. This may reflect the higher amounts paid for workers’ compensation claims, for which providers are reimbursed up to 150 % of the AMA listed fees, which are in turn higher than the government-subsidized fees listed in the Australian Medical Benefits Schedule. Lumbar fusion costs rose to AUD 81,297 at 24 months, which is lower than data from the US at USD 72,000 to 90,000 [11, 22]. A cervical fusion cost AUD 37,291 in this study compared to USD 42,401 in the US, the latter of which included physiotherapy costs [23].

Previous studies have reported reoperation rates of 21 % after lumbar decompression and 23 % following lumbar fusion, similar to our results [22, 24]. A NSW study of 476 workers’ compensation patients found 9.2 % of people underwent revision surgery at 24 months after any lumbar surgery, though this may be an underestimate as it was based on chart review without access to the administrative data now available [13]. Reoperation following cervical fusion was 15 % at two years in our cohort, slightly higher than the 12 % seen in in other studies [23, 25].

Authors of a 2015 systematic review found return to work (RTW) at 40 % following lumbar fusion in the workers’ compensation population, though duration of follow-up was unclear, whereas a more recent study reported 20 % RTW after 2 to 3 years [22, 26]. A smaller study in a workers’ compensation cohort in NSW found RTW after lumbar surgery was 50 %, with only 14 % of patients working at pre-injury duties at 24 months (3 % for lumbar fusion surgery). Further, 78 % of participants were still undergoing treatment with physical therapy and/or opioids at two years [13]. Results were similar in our cohort, with 16 % working at full capacity and 13 % at reduced capacity by two years after lumbar fusion. Prior studies found higher rates of RTW after decompression and after cervical fusion compared to lumbar fusion, similar to the current study.

A strength of this study was the use of a large population-based database covering over 3 million workers annually in NSW over a 9-year period. SIRA is the regulator of workers’ compensation in NSW and we were unable to discern indication for surgery, though spinal fractures and dislocations were excluded in order to restrict our analysis to elective surgery. We were not always able to determine whether any surgery subsequent to the initial spinal surgery occurred in the same spine region due to data limitations, so it is possible that some reoperations occurred at different levels. This study did not have a control group, precluding comparison between our cohort and people with workplace-related back pain who did not receive surgery. Finally, the database does not capture patient-reported outcome measures such as pain, function and quality of life, which would add richness and depth to our results. However the low rates of return to work and high rates of reoperation provide indirect evidence that health-related quality of life is likely undermined over the longer term (i.e., at least for two years) in this cohort.

## Conclusions

This is the first comprehensive study to report on spinal surgery outcomes in a workers’ compensation cohort at a population level in Australia. In the context of the poor evidence base supporting lumbar fusion surgery, the high cost, increasing rates, and the increased likelihood of poor outcomes in the workers’ compensation population, we question the value of this procedure in this setting. Consideration should be given to generating better evidence through comparative studies to better determine the effectiveness of this common and costly procedure, and to using this procedure more judiciously. Further work in this area would benefit from established quality benchmarks.

## Supplementary information

**Additional file 1:**

## Data Availability

The data that support the findings of this study are available from the State Insurance Regulatory Authority of NSW but restrictions apply to the availability of these data, which were used under license for the current study, and so are not publicly available. Data are however available from the authors upon reasonable request and with permission of the State Insurance Regulatory Authority of NSW.

## Abbreviations

NSW: New South Wales
SIRA: State Insurance Regulatory Authority
AUD: Australian dollars
AMA: Australian Medical Association
SD: Standard deviation
IQR: Inter-quartile range
US: United States
USD: United States dollars
RTW: Return to work
