# Supplementary material for: Rates, costs, return to work and reoperation following spinal surgery in a workers’ compensation cohort in New South Wales, 2010–2018: a cohort study using administrative data
Source: BMC Health Serv Res. 2021 Sep 11;21:955. doi: 10.1186/s12913-021-06900-8 (PMC8436510; doi:10.1186/s12913-021-06900-8)

Supplement to: Rates, costs, return to work and reoperation following spine surgery in a workers' compensation cohort in New South Wales, 2010-2018: a cohort study using administrative data

Supplemental Table S1. AMA and SIRA variables used to identify exclusion criteria (fracture, dislocation and traumatic brain injury).

Source	Variable	Value(s)	Description
SIRA	Nature of injury	020<br>(pre-July 2011)	Fracture of vertebral column with or without mention of spinal cord lesion
SIRA	Nature of injury	112<br>(post-July 2011)	Fracture of vertebral column without mention of spinal cord lesion
SIRA	Nature of injury	310<br>(post-July 2011)	Quadriplegia involving spinal cord injury
AMA	Item number	MQ305-375	Treatment of fracture, dislocation or fracture-dislocation (SPINE)
SIRA<br><br>**Both conditions must be met**	Nature of injury	030<br>(pre-July 2011)	Dislocation
	Bodily location of injury	310: Upper back<br>311: Lower back<br>318: Back - other and multiple<br>319: Back - unspecified	
SIRA<br><br>**Both conditions must be met**	Nature of injury	201<br>(post-July 2011)	Dislocation
	Bodily location of injury	319: Back - unspecified	
SIRA	Payment classification	PBI001-PBI005	Brain injury rehabilitation<br>These codes apply to patients admitted to an inpatient Brain Injury Rehabilitation Program (BIRP) unit, a Transitional Living Unit or to Compensable non-inpatient services.

Abbreviations: SIRA=State Insurance Regulatory Agency of NSW; AMA=Australian Medical Association List of Medical Services and Fees

Supplement to: Rates, costs, return to work and reoperation following spine surgery in a workers' compensation cohort in New South Wales, 2010-2018: a cohort study using administrative data

Supplemental Table S2. AMA item numbers used to identify surgery type (fusion or decompression).

Fusion			
LT135	Fusion posterior	MT170	Bone graft, poster-lateral fusion, 1-2
LT145	Fusion posterior	MT180	Bone graft, poster-lateral fusion, >2 l
LT155	Fusion posterior	MT190	Fusion posterior
LT215	Fusion Cervical	MT200	Fusion multi level
MT030	Fusion Scoliosis or kyphosis	MT210	Fusion Anterior 1 level
MT050	Fusion Scoliosis	MT220	Fusion Anterior 1 level - principal
MT055	Fusion Scoliosis or kyphosis	MT230	Fusion Anterior 1 level - assisting
MT080	Fusion Scoliosis	MT240	Anterior multi level
MT090	Fusion Scoliosis, > 4 levels	MT250	Anterior multi level - principal
MT100	Fusion Scoliosis	MT260	Anterior multi level - assisting
MT110	Fusion Scoliosis	MT270	Fusion
MT120	Fusion Scoliosis, congenital	MT280	Fusion - not scoliosis
MT140	Vertebral body, excision, incl. bone graft	MT290	Fusion - not scoliosis
MT145	Vertebral body, disease of, excision an	MT300	Fusion multi level - not scoliosis
MT150	Bone graft, posterior, 1-2 levels	MT310	Fusion multi level - not scoliosis
MT160	Bone graft, posterior, >2 levels		
**Decompression**			
LT045	Single Level	LT195	Cervical
LT055	Single Level	LT205	Cervical
LT065	Single Level	LT255	Thoracic
LT075	Decompression multi level	LT265	Thoracic
LT165	Single Level (Spinal rhizolysis)	LT275	Thoraco lumbar or high lumbar
LT175	Cervical	MT130	Lumbar, 1+ levels
LT185	Cervical	MT230	Fusion Anterior 1 level - assisting

Abbreviations: AMA=Australian Medical Association List of Medical Services and Fees

Supplemental Table S3. AMA and SIRA variables used to identify spine region (cervical, lumbar).

**Cervical**			
Source	Variable	Value	Description
AMA	Item number	LT215	Cervical decompression of spinal cord
		MT342	Cervical artificial intervertebral total disc replacement
		LT175	Cervical decompression of spinal cord
		LT185	Cervical decompression of spinal cord
		LT195	Cervical partial or total discectomy (anterior)
		LT205	Cervical decompression of spinal cord
SIRA	bodily location of injury	210	Neck bones, muscles and tendons
		218	Neck - other and multiple
		219	Neck - unspecified
		620	Head and neck
		660	Neck and shoulder
**Lumbar**			
AMA	Item number	MT320	Lumbar artificial intervertebral total disc replacement
		MT330	Lumbar artificial intervertebral total disc replacement
		MT340	Lumbar artificial intervertebral total disc replacement
		MT130	Percutaneous lumbar partial or total discectomy
		LT275	Thoraco-lumbar or high lumbar anterior decompression
SIRA	bodily location of injury	311	Lower back

Abbreviations: SIRA=State Insurance Regulatory Agency of NSW; AMA=Australian Medical Association List of Medical Services and Fees

Supplemental Table S4. Return-to-work status at 24 months post-surgery, by surgery type

	**Fusion**<br>**N=2,762**	**Decompression**<br>**N=4,279**
Working at full capacity	524 (18.9)	1674 (39.1)
Working at reduced capacity	354 (12.8)	547 (12.8)
Not working	1781 (64.4)	1943 (45.4)
Retired	90 (3.2)	108 (2.5)
Deceased	13 (0.5)	7 (0.2)

* Restricted to surgeries from 2010-2016 to allow 24 months for outcome ascertainment.

Supplement to: Rates, costs, return to work and reoperation following spine surgery in a workers' compensation cohort in New South Wales, 2010-2018: a cohort study using administrative data

Supplemental Figure S1. Reoperation at 24 months by surgery type and spine region.

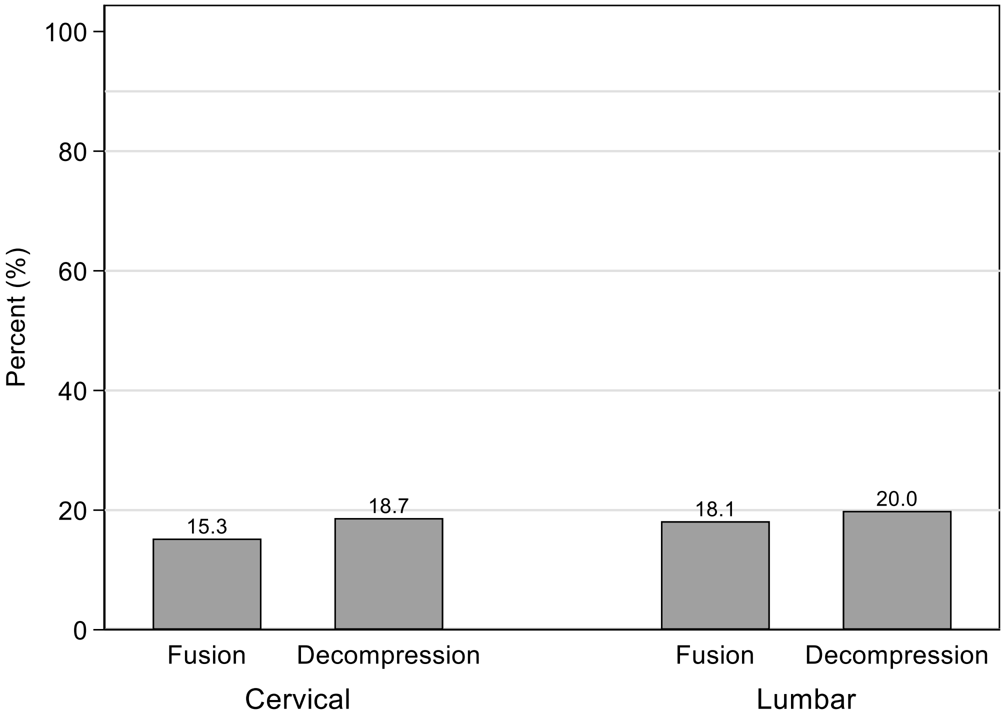

Supplement: Supplementary file 1 — Additional file 1: [file 12913_2021_6900_MOESM1_ESM.pdf]
